# Optimization of Low-Biomass Sample Collection and Quantitative PCR-Based Titration Impact 16S rRNA Microbiome Resolution

**DOI:** 10.1128/spectrum.02255-22

**Published:** 2022-11-15

**Authors:** Benjamin Gregory James Clokie, Ahmed Elsheshtawy, Amaya Albalat, Are Nylund, Allan Beveridge, Chris J. Payne, Simon MacKenzie

**Affiliations:** a Institute of Aquaculture, University of Stirling, Stirling, United Kingdom; b Faculty of Aquatic and Fisheries Sciences, Kafrelsheikh University, Kafr El Sheikh City, Egypt; c Fish Disease Research Group, Department of Biological Sciences, University of Bergen, Bergen, Norway; University of Guelph

**Keywords:** 16S rRNA, complex, equicopy, gill, low-biomass, titration

## Abstract

The major aquatic interface between host and environment in teleost finfish species is the gill. The diversity of this infraclass, high complexity of the organ, and its direct exposure to the surrounding environment make it an ideal candidate for furthering our understanding of the intertwined relationships between host and microbiome. Capturing the structure and diversity of bacterial communities from this low-biomass, inhibitor-rich tissue can, however, prove challenging. Lessons learned in doing so are directly applicable to similar sample types in other areas of microbiology. Through the development of a quantitative PCR assay for both host material and 16S rRNA genes, we tested and developed a robust method for low-biomass sample collection which minimized host DNA contamination. Quantification of 16S rRNA facilitated not only the screening of samples prior to costly library construction and sequencing but also the production of equicopy libraries based on 16S rRNA gene copies. A significant increase in diversity of bacteria captured was achieved, providing greater information on the true structure of the microbial community. Such findings offer important information for determining functional processes. Results were confirmed across fresh, brackish, and marine environs with four different fish species, with results showing broad homology between samples, demonstrating the robustness of the approach. Evidence presented is widely applicable to samples similar in composition, such as sputum or mucus, or those that are challenging due to the inherent inclusion of inhibitors.

**IMPORTANCE** The interaction between the fish gill and surrounding bacteria-rich water provides an intriguing model for examining the interaction between the fish, free-floating bacteria, and the bacterial microbiome on the gill surface. Samples that are inherently low in bacteria, or that have components that inhibit the ability to produce libraries that identify the components of microbial communities, present significant challenges. Gill samples present both of these types of challenges. We developed methods for quantifying both the bacterial and host DNA material and established a sampling method which both reduced inhibitor content and maximized bacterial diversity. By quantifying and normalizing bacteria prior to library construction, we showed significant improvements with regards to the fidelity of the final data. Our results support wide-ranging applications for analyzing samples of similar composition, such as mucus and sputum, in other microbiological spheres.

## INTRODUCTION

The aquatic interface that is provided by the fish gill presents an intriguing model with which to challenge, query, and present theories regarding the interaction between host, microbiome, and environment. Direct contact with the bacterially rich aqueous environment, inherent permeability, and the fundamental physiological role of the fish gill offer important attributes for this organ’s utilization as an important biological model. The gill microbiome also offers promise as a health management tool for high-value finfish aquaculture. Recent studies examining freshwater hatchery rearing (1), amoebic gill disease (AGD) (2), and the interplay of diet and rearing system (3) have all sought to exploit the aforementioned attributes. Intensive culture conditions in commercial aquaculture are fueling the development of noninvasive health management tools that seek to provide accuracy and protect fish welfare (4) with the potential to perform nonlethal longitudinal studies under commercial conditions. Although clearly promising, samples can be both low in biomass and rich in inhibitors, resulting in difficult technical challenges compared to samples rich in bacterial targets, such as stool. Despite progress in other low-bacterial-load samples, such as lung tissue, cancer tumors, eyeballs, and the cervix, there exists limited information regarding how to collect samples and how to optimize library construction to deal with these challenges in aquatic environments.

Method development has been, and will continue to be, a key target for microbiome research (5), and the development of robust and reproducible methods for monitoring microbial diversity of different niches provides a major challenge (6). Errors and biases resulting from extraction kits (7), reagent contamination (8), and library preparation methods and parameters used for amplification have been well documented (9), and solving such problems is required for the advancement of microbiome research. Next-generation sequencing has facilitated the development of two major approaches for such analysis: metagenomic analysis, which allows the total genomic landscape to be identified, enabling high-resolution bacterial identification to a species level, and amplification of regions of the 16S rRNA gene and subsequent sequencing of the resulting amplicons, which offer a more cost-effective approach. Amplicon analysis is performed through the examination of variations in the hypervariable regions of the 16S rRNA gene by using operational taxonomic units (OTUs) or amplicons sequence variants. Optimized primers, primarily centered on the V3 and V4 regions, provide the broad coverage required (10). A common problem to both techniques is the high representation of host DNA relative to bacterial DNA, and this is further exacerbated by low-biomass samples, such as with mucous membranes. Birlanga et al. (2) demonstrated this when they reported that salmon DNA represented three-quarters of the total returned reads when they analyzed salmon mucus in fish infected with AGD.

Minimizing host reads can be achieved either prior to or post-DNA extraction through biochemical manipulation (11). Preextraction approaches include (i) host cell lysis, exploiting the weaker structure of the host cell membrane compared to peptidoglycan-rich bacterial cell walls, (ii) the removal of exposed DNA using enzymes, or (iii) propidium monoazide, which leaves bacterial cells intact (11). Preextraction methods have improved the microbial sequencing in many human sample types (12–14); however, a potential loss of bacterial DNA and bias toward Gram-positive bacteria have also been reported (15). Postextraction methods, such as methylated nucleotides, hybridization-based depletion for host DNA with CRISPR/Cas9, or magnetic beads coupled to blocking primers (16, 17), are available. Examples in fish are limited; however, the microbial DNA of black mollies (*Poecilia latipinna*) was successfully enriched via depletion of host methylated DNA using MBD-Fc beads to increase sequence coverage (17). Again, methylation-based approaches have been shown to bias samples, with particular reference to microbes with AT-rich genomes. Two further considerations for such depletion methods are the cost and the difficulty in performance at genome scale (11, 18).

An alternative to postsample nontarget DNA depletion is the development of protocols that minimize collection of host material and maximize microbial recovery. The fish gill is the major respiratory organ and is surrounded by a mucus-rich layer that provides an extensive microbial niche. Recent gill microbiome studies have utilized terminal samples, such as gill fragments or entire gills (19–22), the gill filament without arch (23), or scraped gill mucus (2, 24), with results showing the potential of the gill as an informative biological model. Swabs (1, 25) offer an alternative noninvasive approach; however, data recovery must be shown to be robust, capturing the high spatial and temporal heterogenicity of real-world situations (2, 26). Solubilization of membrane proteins and associated matrices using surfactants such as Tween and NP-40, which have differing levels of aggressiveness, is a commonly adopted approach in molecular biology to study extracellular surfaces. Practical considerations of *in situ* sample collection and postcollection sample processing (1, 3, 27) must be considered and optimized. Achieving such goals offers the potential for gill microbiome analysis to be used not only as an integral part of a cohesive health management strategy but also as an exciting model with which to explore current themes in microbiome research, such as the holobiont concept and neutrality (28).

## RESULTS

**FIG 1:**
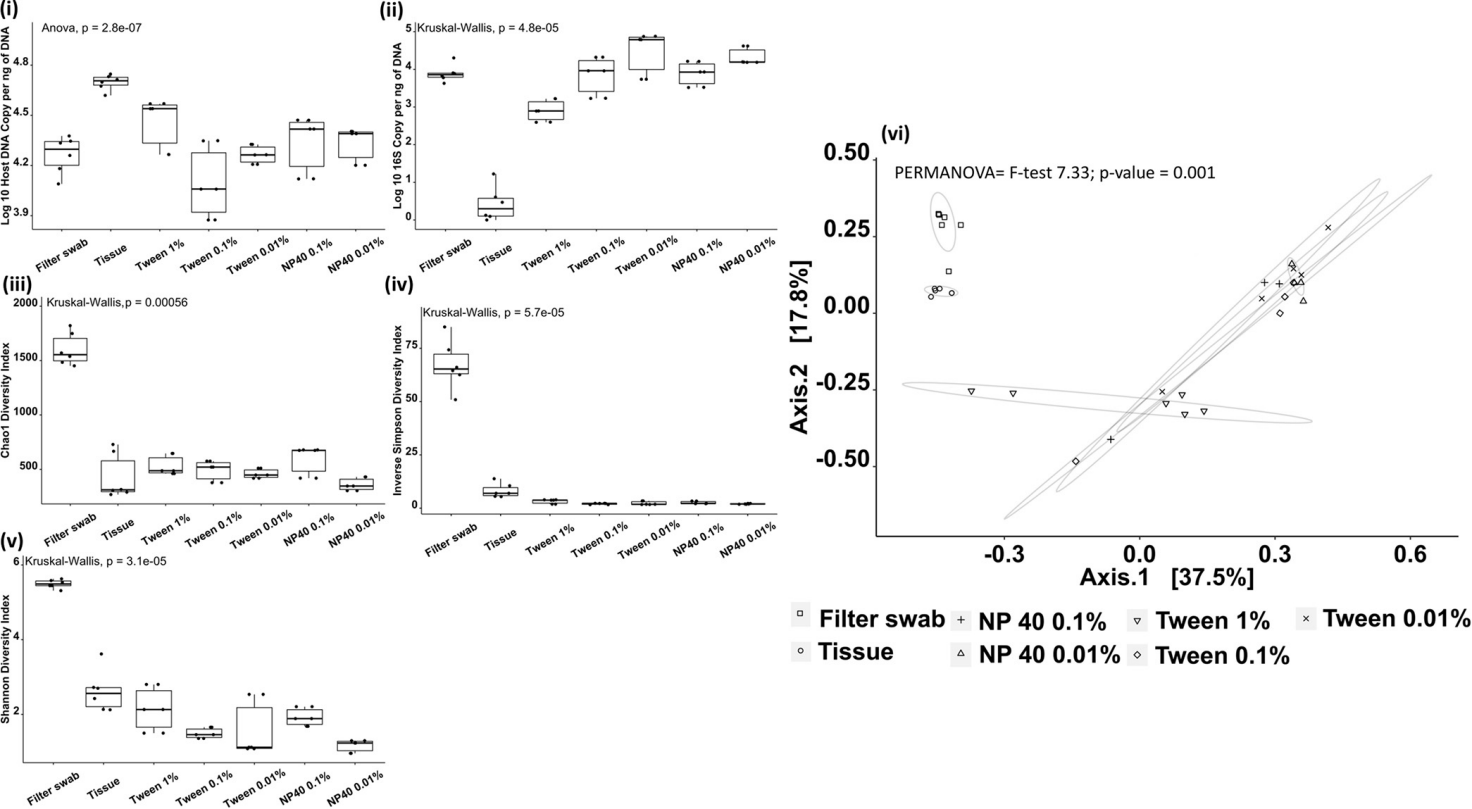
Comparison of a range of sample approaches on fish gills. (i and ii) Host DNA (i) and bacterial 16S rRNA (ii) were measured using Abs-qPCR, with significantly more host material and significantly fewer 16S copies in tissue compared to all other approaches (BH-corrected *P*, 0.00002 to 0.009). (iii, iv, and v) Chao1 (iii), inverse Simpson (iv), and Shannon diversity (v) indices were calculated based upon the V4 region obtained by 16S amplicon sequencing. For each index metric, significantly higher values were recorded for the filter swabs versus other approaches (BH-corrected *P*, 0.008 to 0.045). A PCoA plot based on a Bray-Curtis similarity matrix of microbial communities (iv) comparing groups using PERMANOVA showed significant changes in grouping (*F* = 7.33, *P* = 0.001). Circles enclosing data points are intended to be visual guides.

Our sampling approach had a clear and measurable impact on 16S rRNA gene recovery, with gill tissue yielding significantly fewer copies of 16S rRNA genes (Kruskal-Wallis *P* = 4.793e−05) and significantly more host DNA (analysis of variance [ANOVA] *P* value = 2.78e−07) than all other methods (Fig. 1). A dose-response pattern regarding hemolysis was also identifiable, as higher concentrations of surfactant resulted in discoloration of the wash through the rupturing of gill tissue (data not shown), and these observations were supported with host DNA recovery being significantly higher for Tween 20 at a 1% concentration compared to that with 0.1% (pairwise *t* test *P* = 1.41e−4) and 0.01% Tween 20 samples (pairwise *t* test *P* = 3.24e−2). Following extraction and quantification, libraries normalized by copy number demonstrated a clear difference between the gill filter swab and all other methods (see Table S1 in the supplemental material). Despite a positive yield from the surfactant wash in terms of 16S rRNA amplicon recovery, this was not reflected in either Chao1 diversity, a metric that accounts for singletons and the resulting impact of these on community structure, or the inverse Simpson or Shannon diversity indices, which reflect the evenness of bacterial species present across a sample, identified postsequencing. A principal-coordinate analysis (PCoA) plot based on a Bray-Curtis similarity matrix of microbial communities highlighted clustering for the sampling approach, which had a demonstrable impact on the data recovered, with filter swab samples tightly grouped (permutational multivariate ANOVA [PERMANOVA] overall *F* = 7.33, overall *P* = 0.001). All wash samples were observed to group together regardless of concentration and separate from both the filter swabs and whole-tissue samples. Comparisons between filter swabs and all washes showed significant differences (*P* values, 0.006 to 0.009), as did whole tissue and all washes (*P =* 0.006) (Table S2). In addition, differences between the filter swabs and the tissue samples were also observed (*P* = 0.006).

**FIG 2:**
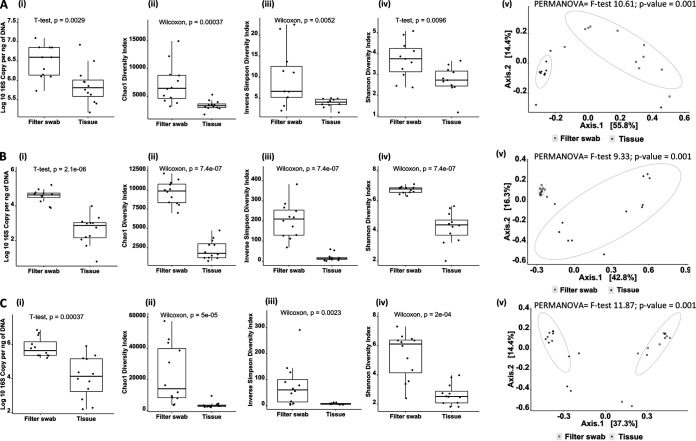
Comparison of filter swabs versus gill tissue as a sampling approach in three different fish species: *Mugil capito* (gray mullet) (A), *Oreochromis niloticus* (Nile tilapia) (B), and *Salmo salar* (Atlantic salmon) (C). Column (i) reports the copy number of 16S per nanogram of DNA, measured using Abs-qPCR. Columns (ii) to (iv) show the Chao1, inverse Simpson, and Shannon diversity index results obtained from sequencing the V4 region of the 16S rRNA subunit with pairwise comparison values (for nonnormal distribution, Wilcoxon test; for normal distribution, *t* test). Colum (v) shows the PCoA plot based on a Bray-Curtis similarity matrix of microbial communities for each comparison and overall PERMANOVA results. Circles enclosing data points are intended to be visual guides.

Complexity surrounding filter recovery from syringe filters influenced the decision to proceed with filter swabs instead of washes, despite 16S amplification being comparable (no sequence data were available at this point). Comparison of methodologies using fish of different species and culture conditions yielded data of high similarity to initial findings. In all instances, significantly higher amplification of 16S rRNA genes was achieved in filter swabs versus whole tissue, and after equicopy libraries were constructed both diversity and evenness were significantly greater using swabbing (Fig. 2). As in the initial trial, sampling method had a strong influence over sample similarity observed via PCoA (PERMANOVA, gray mullet, overall *F* = 9.91, *P* = 0.001; PERMANOVA, Nile tilapia, overall *F* = 9.33, *P* = 0.0009; PERMANOVA, Atlantic salmon, overall *F* = 11.87, *P* = 0.0009).

**FIG 3:**
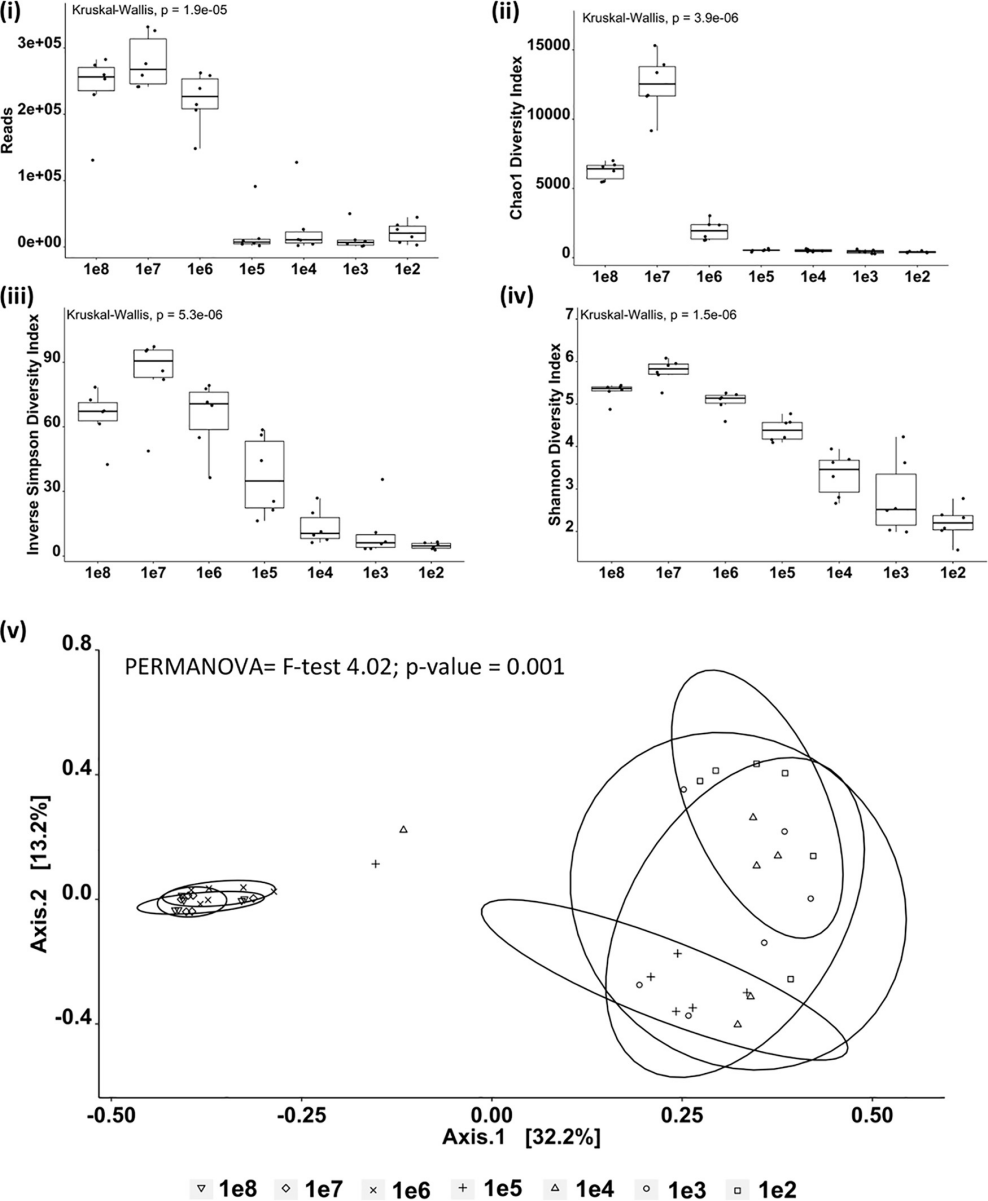
Water samples collected from the same site in the Nile Delta were normalized to 1e8 bacterial 16S rRNA copy numbers prior to a serial dilution from 1e8 to 1e2. Each dilution was sequenced using V4 primers for 16S rRNA. (i) Reads obtained following sequencing; all values below 1e5 were significantly lower than 1e8 to 1e6 (BH-corrected *P* = 0.04) (all *P* values are reported in Table S3 in the supplemental material). (ii, iii, and iv) Chao1 diversity (ii), inverse Simpson (iii), and Shannon (iv) diversity indices all exhibited significantly lower values in all samples below 1e6 (corrected *P*, 0.048 to 0.005). (v) PCoA plot based on a Bray-Curtis similarity matrix of microbial communities calculated from initial 16S concentrations shows significant differences between the 1e8, 1e7, and 1e6 groups against all other groups (corrected *P*, 0.010 to 0.006). Circles enclosing data points are intended as visual guides only.

Sample richness based upon the presence of identifiable OTUs was determined through serial dilution of six water samples with high initial 16S amplification (Fig. 3). Samples were normalized to 1e8 before a dilution series from 1e8 to 1e2 was sequenced. Results showed a significant drop in the number of reads obtained below 1e6, with *P* values ranging from 0.048 to 0.005 (all *P* values are reported in Table S3). Reducing input material gave rise to a sudden reduction in the Chao1 diversity score below 1e6, with 1e8, 1e7, and 1e6 Chao1 scores significantly different from lower-input libraries (Benjamini-Hochberg [BH]-corrected *P =* 0.003). As input concentration decreased, both inverse Simpson and Shannon diversity matrices showed dose-related reductions, with diversity decreasing at each concentration. A PCoA and a subsequent PERMANOVA test showed significant differences between the groups (overall *F* = 7.73, *P* = 0.001), with from 1e8 to 1e6 all clustering and showing no significant differences between them (BH-corrected *P* value range, 0.009 to 0.010) (Table S3).

**FIG 4:**
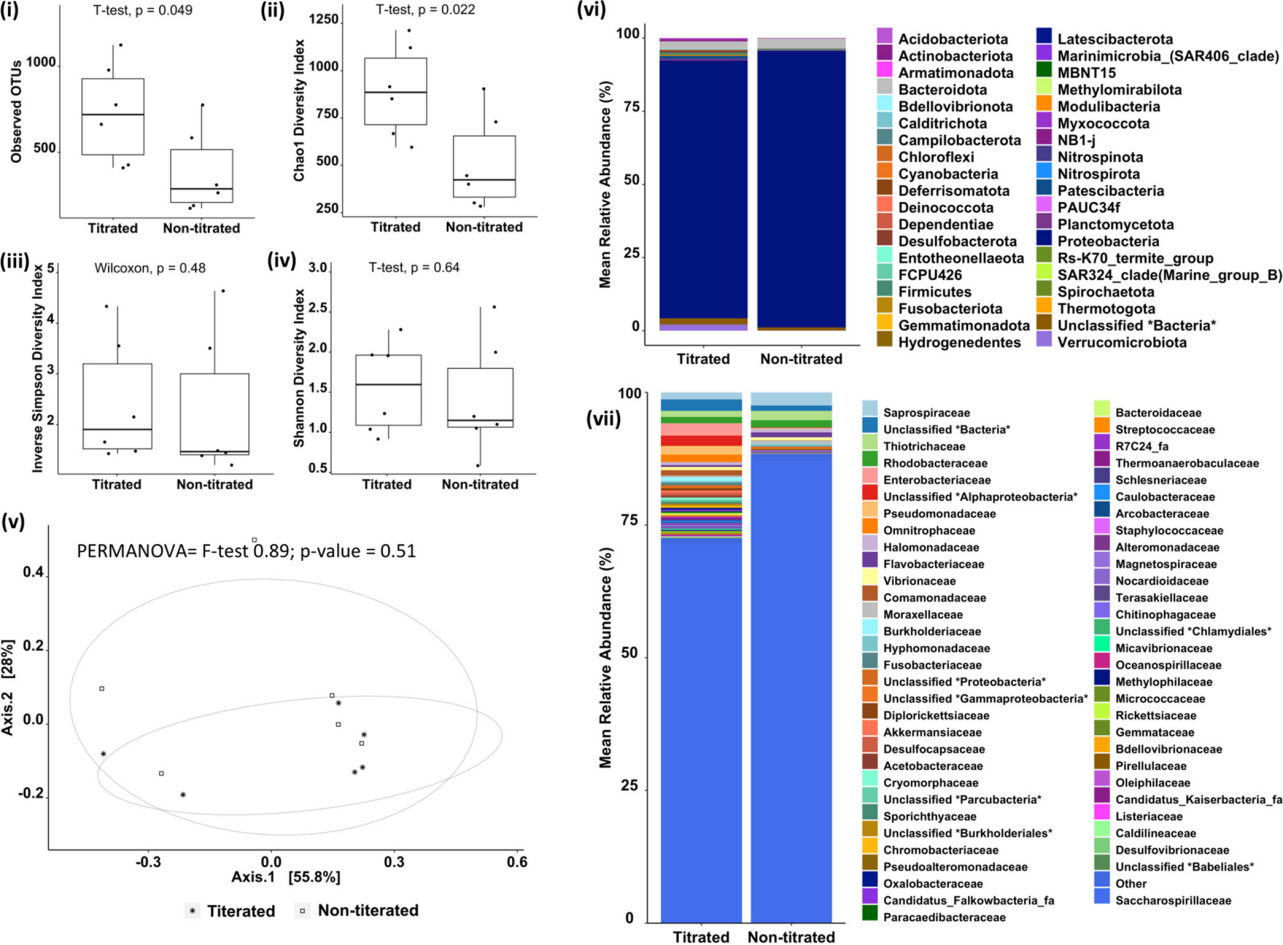
Comparison of libraries constructed from samples collected from the gills of Atlantic salmon using a filter swab. Initial input DNA of either 20 ng or DNA equivalent to 1e6 copies of 16S, as measured using Abs-qPCR, was used prior to amplification of the V4 16S rRNA region. (i to iv) Observed OTUs (i), Chao1 (ii), inverse Simpson (iii), and Shannon diversity (iv) index values are presented with statistical tests applied according to data distribution (for normal data, *t* test; for nonnormal data, Wilcoxon test). (v) PCoA plot based on a Bray-Curtis similarity matrix of microbial communities with overall PERMANOVA values shows grouping of the samples (*F* = 0.89; *P* = 0.51). Circles are intended as a visual guide. (vi and vii) Changes in mean relative abundance of phyla (vi) and family (vii) are shown by colors representing the different bacteria.

Construction of libraries based on initial DNA concentration failed to account for the bacterial biomass within the sample. Comparison of the same samples sequenced by DNA concentration as opposed to 16S copy number are presented in Fig. 4 and show the impact that this had upon the number of observable OTUs. Differences were reflected in Chao1 diversity; however, the inverse Simpson and Shannon diversity indices did not reflect these differences, confirming that observations had stemmed from initial start material as opposed to any technical issue. The clustering of the samples via PCoA showed that overall samples did not cluster separately (PERMANOVA *F* = 0.89; *P* = 0.51); however, clear differences were observable when we visually examined the phyla and families.

## DISCUSSION

Central to developing the fish gill as a model within the context of evolutionary biology, the holobiont, neutrality, or for monitoring health status for commercial stock management is the ability to collect and process samples in a repeatable, robust manner. Low-biomass samples (29) and samples intrinsically rich in inhibitors, such as those containing blood components (30), are problematic for achieving sufficient material for amplicon sequencing. The development of the absolute quantitative PCR (Abs-qPCR) assay described to quantify 16S gene copy number in addition to host DNA provided an essential tool for determining an optimized sampling method based upon empirical recovery. In contrast to several published studies utilizing gill tissue, screening with qPCR highlighted significant challenges in achieving amplification across all four species examined. Similar results were observed with whole gills washed using high concentrations of surfactant, where observable hemolysis of gill tissue occurred. Reduced amplification may be explained either by low biomass of bacteria on the gills or through an inhibitory effect of host material. PCR inhibitors in samples containing blood are well described (31), and testing 16S rRNA copy numbers extracted from a pure bacterial culture with salmon gill DNA confirmed an inhibitory effect below1e4. Although successful removal of difficult inhibitors has been reported using the PowerClean DNA clean-up kit (Qiagen, previously MoBio) (32, 33), we failed to achieve any amplification (data not shown).

Both mechanical and chemical sloughing of the mucus significantly improved 16S rRNA recovery in comparison to whole-tissue samples. Interestingly, significantly greater abundance and diversity of bacteria were recorded via gill filter swabs as opposed to gill washes at all concentrations. Observed statistical differences in diversity were supported by significantly different taxa presented within the heat trees (see Fig. S1 in the supplemental material). The ability of a filter to capture a wide area of both sides of the gill arch may help achieve this. Surfactant washing, however, fails to achieve the same, suggesting that ensuring sufficient bacteria are removed from the surface while maintaining the underlying gill structure is a difficult balance. As previously discussed, overly aggressive washing induces inhibition through lysis of the gill structure, whereas nonaggressive concentrations fail to remove sufficient material. The physical action of the polycarbonate filter likely helps to dislodge resident bacteria while maintaining the gill structure below. Sonification has been suggested by Birlanga et al. (34) to assist with this delicate balance; however, the simplicity of using a filter membrane provides several benefits regarding welfare and repeatability. Importantly, results appeared to be robust between the aquatic environments of freshwater (i.e., rainbow trout), brackish water (i.e., gray mullet and tilapia), and salt water (Atlantic salmon), in both cold (rainbow trout and Atlantic salmon) and hot (gray mullet and tilapia) environments, as seen via the clustering-of-samples technique using PCoA. The nonaggressive nature of using filter swabs as prescribed here allows the capture of data with suitable resolution of diversity and abundance for sampling the fish gill microbiome.

While pertinent to finfish, the problem regarding low-biomass samples is not exclusive. Low-copy-number samples, such as those from sputum (35) or mucosal surfaces (36), can present significant challenges for library construction, as demonstrated via the serial dilution of water samples from 1e8 to 1e2. Accurate observation of OTU diversity, alpha and beta characteristics, rely upon sufficient copies with which to derive relative abundance statistics. A reduction in the initial number of 16S copies dramatically changes the structure of the microbial community, as evidenced below 1e5 with concurrent reductions in diversity indexes. Screening samples prior to indexing and sequencing can also help to reduce unnecessary costs, as gross DNA concentration fails to account for the proportion of 16S copies (Table S5). In a recent trial in our laboratory, screening showed that only 26% of the samples collected met a threshold above 1e5 (Fig. S2), emphasizing the highly variable nature of low-biomass samples. Such variability can be accounted for through the adjustment of starting material for library construction (Fig. S3). Prior experience of quantification can help to inform future sampling events, ensuring sufficient samples are collected and informing experimental design.

An important aspect of exploring microbial communities using sequencing technology is the ability to circumvent difficult conventional microbiology culture techniques and problems surrounding the growth of unculturable organisms. Despite obvious potential bias toward culturable bacteria, assimilation of data with available functional information allows progression from merely descriptive studies, as long as the true bacterial populations are captured. Constructing libraries with the titrated initial 16S concentration provides an important step in achieving this, as the domination of highly represented bacterial species seen in samples normalized to total DNA is negated, OTU counts and Chao1 are significantly increased, and examination of bacteria at both the phylum and family levels exhibits visible differences. The titrated versus nontitrated samples presented in this study resulted from the selection of samples with high initial 16S copy numbers in order to numerate the comparison. In a random selection of samples, however, it is likely that some samples with a very low copy number will be included and the observable difference will be much larger. Biological samples are inherently variable, and where 16S rRNA copy numbers are impinged either by low biomass or the presence of inhibitors, quantification and titration facilitate the construction of broadly equivalent libraries between samples. Studies that fail to account for these differences may incorrectly reject hypotheses, as was the case for Slinger et al. (37), who reported no significant differences between sampling approaches. Community analysis is performed using complex tools derived from ecology that require considerable knowledge for meaningful interpretation. Appropriate considerations and limitations must be applied to the analysis of complex data sets with particular pertinence to analyses when the totality of the bacterial population is unknown. Amplicon sequencing is stochastic in nature. Resolution of technical variance driven by accurate initial library construction helps to reduce experimental noise for downstream analysis, improving the accuracy of statistical models. Here, we propose that the adoption of quantification can provide a considerable step toward deriving robust data sets from complex, low-biomass environments.

Data presented in this study outlined the importance of minimizing host genomic material in samples that are prone to blood contamination, such as fish gills. We have presented evidence that screening samples prior to library construction ensures that expensive library construction and sequencing procedures are appropriately applied and offer significant benefits. Finally, we have demonstrated the impact that titration of the 16S gene prior to library construction can have for deriving robust and reproducible data for low-biomass samples.

## MATERIALS AND METHODS

### Ethics statement.

This study was carried out in accordance with the UK Animal Scientific Procedures Act. The study protocol was approved by the University of Stirling Animal Welfare and Ethical Review Body [AWERB (19/20) 63 and AWERB (18/19) 196] and Norwegian Animal Research Authority (NARA) in 2019 under the identification code 18259.

### Evaluation of different sampling approaches for gill microbiome analysis.

Assessment of sampling methods using gill filter swab, gill wash, and gill tissue was performed on rainbow trout (Oncorhynchus mykiss) gill samples collected from 6 rainbow trout (mean weight 495.45 ± 170.86 g; fork length [FL] 32 ± 2.9 cm) (means ± standard deviations [SD]) sourced from the NBFRU (Institute of Aquaculture, University of Stirling, Scotland). A lethal overdose of the anesthetic tricaine mesylate (MS-222; 100 ppm) was followed by severance of the caudal peduncle. The operculum and the first gill arch were lifted using sterile forceps and Nuclepore track-etched membrane filters (Whatman International Ltd., United Kingdom) held on opposing sides of the second gill arch, and the operculum was released. For 30 s, gentle pressure was applied on the operculum, and the filter removed. The filter swab was immediately snap-frozen and stored at −80°C until DNA extraction. Gill wash samples were collected by removing the opposing gill arches and placing them individually into 10 mL of either Tween 20 (Thermo Scientific, USA) at 1%, 0.1%, or 0.01% or NP-40 (Thermo Scientific, USA) at 0.1% or 0.01%. A section of gill filament tissue was excised using sterile scissors and snap-frozen.

### Comparison of tissue versus filter swabs in different species.

Twelve Nile tilapia (Oreochromis niloticus; 309.8 ± 38.4 g, FL 24.25 ± 0.98 cm) and 12 gray mullet (Mugil capito; 162.4 ± 24.1 g, FL 25 ± 2.3 cm) were collected from a commercial semi-intensive polyculture fish farm in the Egyptian Nile delta. Pond parameters (35°C water temperature, 0.12 mg/liter NH_3_, dissolved oxygen 6.5 to 7.5 mg/liter). Fish were sacrificed by immersion in ice before a gill filter swab and gill tissue were collected as described above. Due to the basic facilities available, samples were stored directly in 1 mL Longmire's buffer (0.1 M Tris, 0.1 M EDTA, 10 mM NaCl, 0.5% [wt/vol] SDS [Thermo Scientific, USA]) (38), frozen, and stored at −20°C until DNA extraction.

Atlantic salmon (*Salmo salar*) samples were obtained from Industrial and Aquatic Laboratory (ILAB) at the University of Bergen, Norway. Gill filter swabs and gill tissue were collected as previously described from 12 postsmolt Atlantic salmon (246.18 ± 28.6 g, FL 27.5 ± 1.01 cm). Samples were stored directly in 1 mL Longmire's buffer (38), frozen, and stored at −20°C until DNA extraction.

### Bacterial DNA quantification (Abs-qPCR).

16S rRNA gene quantification for all samples reported in this study were assessed using a TaqMan Abs-qPCR assay targeting the V3-V4 region with an amplified target of ~463 bp (Table S6). V3-V4 plasmid standards were produced by cloning the target from a selection of DNA extracted from five aquatically relevant bacterial species: Aeromonas hydrophila NCIMB 9240, Edwardsiella ictaluri NCIMB 13272, Pseudomonas aeruginosa ATCC 27853, Vibrio anguillarum NCIMB 6, and Yersinia ruckeri NCIMB 2194. PCR product was purified using NucleoSpin gel and PCR clean-up (Macherey-Nagel, Germany) according to the manufacturer’s instructions and ligated into pGEM-T Easy vector systems (Promega, United Kingdom), with XL1-Blue competent cells (Agilent Technologies, USA) following the manufacturer’s protocol. Sanger sequencing of the T7 promoter (Eurofins Genomics, United Kingdom) was performed, and Clustal Omega multiple-sequence alignment software was used to align primers prior to performing an NCBI Blast analysis on product sequence. Insert size was 463 and belonged to Yersinia ruckeri. Calculations of copy numbers per microliter of plasmid DNA were performed and dilutions from 1 × 10^7^ to 1 × 10^1^ copy/μL were made using nuclease-free water. Quantification was performed in 20-μL reaction volumes (10 μL 2× SensiFAST Probe Lo-ROX mix [Bioline, United Kingdom]; 0.5 μL of 10 mM F and R primers [final concentration, 400 nM]; 2 μL of plasmid dilution; 0.1 μL of V3-V4 probe [final concentration, 100 nM]; 7.1 μL nuclease-free water). PCR conditions of 95°C for 10 min followed by 40 cycles of 95°C for 30 s and 60°C for 1 min were replicated on five independent runs, and mean threshold cycle (*C_T_*) values were plotted against log copy number to generate a standard curve. Sample analysis was performed in triplicate (20 ng concentration/reaction mixture), and copy numbers per 1 ng of DNA were determined and used for calculations of starting material for library preparation.

### Host genomic DNA quantification.

Primers spanning the intron-exon boundary of the *Salmo salar* claudin 18 gene (accession number NC_027309.1) fragment size of 241 bp and Oncorhynchus mykiss sodium/potassium-transporting ATPase subunit alpha-1 (accession number NC_035095.1) fragment size 173 bp (Table 1) were designed for quantifying host genomic DNA via absolute qPCR. Gene targets were amplified from gill samples and cloned as per the 16S rRNA gene target and plasmid DNA extracted, quantified, and used to build a standard curve from 10^7^ to 10^1^ copies. qPCR was performed in a 20-μL reaction mix (10 μL 2× Luminaris Color HiGreen qPCR master mix [ThermoFisher Scientific, United Kingdom], 0.5 μL of forward and reverse primers, 2 μL of plasmid dilution, and 7 μL nuclease-free water). PCR conditions of 95°C for 10 min were followed by 35 cycles of 95°C for 30 s, 60°C for 30 s, and 72°C for 30 s. Melting curve analysis was performed from 60°C to 90°C with 0.1°C increments per second to evaluate the qPCR product specificities. Five independent runs were conducted, and mean *C_T_* values were plotted against log copy number to generate the standard curve. Samples were run in triplicate using 20 ng DNA per reaction mixture for quantification of the host genomic load.

### DNA extraction.

Sample data presented in Fig. 1 were extracted using an EZNA tissue DNA kit (Omega Bio-Tek Inc., USA) according to the manufacturer’s protocol. Prior to extraction, gill wash samples were defrosted on ice, and 10 mL wash buffer was filtered through a 0.2-μm-pore-size syringe filter (Whatman International Ltd., United Kingdom). The filter was extricated and used for DNA extraction. Gill filter swabs were extracted directly from the filter. Whole tissue was homogenized using a bead beater and lysis kit buffer. Following lysis, samples were bead beaten for 1 min. To increase the efficiency of DNA extraction from Gram-positive bacteria, with their prominent feature of thick peptidoglycan cell walls, samples were heated to 95°C for 10 min as per Knudsen et al. (7). Standard kit instructions were followed for DNA eluted from columns using 50 to 200 μL elution buffer. DNA purity and concentration were evaluated using a Nanodrop ND-1000 spectrophotometer (ThermoFisher Scientific, United Kingdom) and Qubit 2 fluorometer (Invitrogen, USA). Sample data presented in Fig. 2 to 4 were extracted as described above, but we used a Tris-EDTA lysis buffer following the methods described by Longmire et al. (38).

### Impact of starting 16S rRNA concentration on microbiome recovery and diversity.

Determination of the impact of the starting 16S rRNA concentration on recoverable reads and sample diversity was performed using six water samples collected from the same site at the same time as the gill samples from tilapia and gray mullet from the Nile Delta. Samples were double filtered through 0.4-μm- and 0.2-μm-pore-size Nuclepore filters (Whatman International Ltd., United Kingdom). Filters were stored in Longmire’s solution prior to DNA extraction directly on the filter. 16S rRNA copy number was assessed though qPCR, and a serial dilution was performed for a total count from 10^8^ to 10^2^ for each sample.

### Comparison of bacterial communities between samples normalized to 16S rRNA copy number and DNA concentration.

DNA from the same six gill filter swabs selected from the ILAB trial was used to construct libraries based on either 20 ng total initial DNA or a 1e6 copy number as measured by qPCR.

### Preparation of amplicon library for Illumina sequencing.

With the exception of the 20-ng initial DNA samples, all the samples within each data set were normalized to equicopy 16S rRNA according to the qPCR assay. In addition to the 16S rRNA libraries, negative sequencing controls, no-template controls, and a control Institute of Aquaculture microbiome standard were constructed. Potential maximum copy numbers were determined according to the sample set, and libraries were constructed reflecting lowest available copies.

### Library construction.

A first PCR was performed to amplify a fragment of the V4 region of the bacterial 16S rRNA gene using a forward and reverse primer cocktail with adaptors (Table S6). All samples were amplified in triplicate. A 10-μL end-volume PCR (5 μL 2× NEBNext Ultra II Q5 [New England Biolabs, United Kingdom], 0.4 μL V4 forward primer [0.2 μM] and 0.4 μL V4 reverse primer [0.2 μM] cocktail, and 4.2 μL sample). PCR conditions were 98°C for 2 min, 25 cycles of 98°C for 15 s, 54°C for 30 s, and 65°C for 45 s, and a final extension step at 65°C for 10 min. Triplicate first PCR products were pooled and examined in a 1.5% agarose gel to observe a product size of ~312 bp including Illumina adapter sequences. Amplicons were purified using the AxyPrep Mag PCR clean-up kit (Axygen Biosciences, USA) with a modified 1:1 volume of PCR product with AxyPrep beads. The manufacturer’s protocol was followed, and DNA was eluted into 15 μL of elution buffer (EB) buffer (Qiagen, Germany). Nextera XT index kit sets A, B, C, and D (Illumina, USA) were bound to the 5′ and 3′ ends. Eight cycles of indexing PCR were performed with the same conditions as the first PCR, in a total volume of 30 μL (15 μL of 2× NEBNext Ultra II Q5, 10 μL of purified first PCR product, Nextera XT Index 1 [i7] and 2 [i5] primers [2.5 μL each]). Next, 1.5% agarose gel electrophoresis confirmed the expected indexed size of ~381 bp. Quantification of DNA was performed with the Qubit dsDNA HS assay kit (Thermo Fisher Scientific, USA). The final library pool for sequencing utilized 2 ng of material from each sample. Concentration and cleanup were conducted using an AxyPrep Mag PCR clean-up kit and eluted using Tris-EDTA buffer (Thermo Fisher Scientific, USA). Final pool concentration was measured twice with the Qubit high-sensitivity DNA kit on a Bioanalyzer 2100, giving a concentration of ≥0.8 ng/μL. Sequencing was performed using an SP4 flow cell on an Illumina Novaseq 250PE system by Novogene (Cambridge, United Kingdom). Reads returned ranged from 1,127,062 to 509 with a median value of 203,370.

### Statistical analysis.

All raw data was processed through the bioinformatic pipeline Mothur (v1.44.2) (39). The MiSeq standard operating procedure was followed using a minimum sequence length of 274 bp (40). Shared community and genus-level phenotyping files were created via the SILVA SSU database (version 138) (41) using operational taxonomic units binned via a 97% identity for sequence alignment (silva.nr.v138.regionV4.align). Statistical analysis was performed in R Studio (v.1.2.5042). The phyloseq package (version 1.32.0) (42) was used to calculate alpha diversity, and a Shapiro-Wilks test was used to verify homogeneity of variance of the alpha diversity estimates prior to testing between groups. Normally distributed data were analyzed using an ANOVA, and pairwise analyses of ANOVA results were made using Tukey’s honestly significant difference test. Kruskal-Wallace test was used for nonnormal data, and pairwise comparisons were made using a Wilcoxon rank sum test with *P* values adjusted using the Benjamini-Hochberg correction (43). Statistical analysis was performed using the rstatix package (version 0.6.0) (44). Bray-Curtis pairwise distances were utilized for the comparison of beta statistics using the package Vegan (version 2.5.6) (45), and visualization was performed using nonmetric PCoA. Group variance was tested using a nonparametric permutational multivariate analysis of variance (PERMANOVA) with differences considered significant with corrections made for multiple comparisons using the Benjamini-Hochberg correction when the adjusted *P* value was <0.05. All figures were produced using ggplot2 (version 3.3.5) (46). Relative taxa abundance comparisons were performed using differential heat trees showing the log_2_ fold change in taxa abundance through the Metacoder package (version 0.3.4) (47). Between-group taxa comparisons were made using a Wilcoxon rank sum test with a *P* threshold of <0.05 set after correction was made for multiple comparisons using the Benjamini-Hochberg correction.

### Data availability.

Data were submitted to the sequence read archive under accession number PRJNA847066.
