# Supplementary material for: Optimization of Low-Biomass Sample Collection and Quantitative PCR-Based Titration Impact 16S rRNA Microbiome Resolution
Source: Microbiol Spectr. 2022 Nov 15;10(6):e02255-22. doi: 10.1128/spectrum.02255-22 (PMC9769501; doi:10.1128/spectrum.02255-22)
Supplement: Supplemental file 1 — Tables S1 to S6 and Fig. S1 to S3. Download spectrum.02255-22-s0001.pdf, PDF file, 1.5 MB [file spectrum.02255-22-s0001.pdf]

Supplementary Table S1. Table showing Benjamini Hochberg corrected Wilcoxon p-values for alternative sampling approaches for the host DNA, 16S rRNA copy number, Chao1, Inverse Simpson and Shannon presented in Figure 1 (i) – (v).

**Comparison 1**	**Comparison 2**	**Host DNA**	**16S**	**Chao1**	**Inverse Simpson**	**Shannon**
Filter Swab	Tissue	**0.000**	**0.009**	**0.015**	**0.009**	**0.008**
Filter Swab	Tween 1%	**0.032**	**0.009**	**0.015**	**0.009**	**0.008**
Filter Swab	Tween 0.1%	0.053	0.633	**0.015**	**0.009**	**0.008**
Filter Swab	Tween 0.01%	0.995	0.28	**0.015**	**0.009**	**0.008**
Filter Swab	NP40 0.1%	0.435	0.809	**0.015**	**0.009**	**0.008**
Filter Swab	NP40 0.01%	0.435	0.052	**0.015**	**0.009**	**0.008**
Tissue	Tween 1%	**0.009**	**0.009**	0.416	**0.009**	0.416
Tissue	Tween 0.10%	**0.000**	**0.009**	0.416	**0.009**	**0.008**
Tissue	Tween 0.01%	**0.000**	**0.009**	0.416	**0.009**	0.090
Tissue	NP40 0.1%	**0.000**	**0.009**	0.189	**0.009**	0.045
Tissue	NP40 0.01%	**0.000**	**0.009**	0.809	**0.009**	**0.008**
Tween 1%	Tween 0.10%	**0.032**	**0.009**	0.809	0.174	**0.045**
Tween 1%	Tween 0.01%	0.053	**0.009**	0.189	0.051	0.163
Tween 1%	NP40 0.1%	**0.000**	**0.009**	0.416	0.412	0.808
Tween 1%	NP40 0.01%	0.172	**0.009**	**0.015**	0.174	**0.008**
Tween 0.1%	Tween 0.01%	0.053	0.163	0.416	0.808	0.416
Tween 0.1%	NP40 0.1%	**0.009**	0.809	0.189	0.412	**0.008**
Tween 0.1%	NP40 0.01%	0.435	0.163	0.061	0.808	**0.008**
Tween 0.01%	NP40 0.1%	**0.009**	0.163	0.189	0.412	0.416
Tween 0.01%	NP40 0.01%	0.435	0.435	0.061	0.412	**0.008**
NP40 0.1%	NP40 0.01%	0.995	0.163	0.061	0.174	**0.008**

Supplementary Table. S2 Table showing BH corrected p-values for comparisons between alternative sampling approaches for the data presented in Figure 1 PCoA

Fish Species	Statistical test	Comparison	Corrected p-value (Benjamini-Hochberg)
Rainbow trout	PERMANOVA	Different sampling approaches	P=0.001 , R^2= 0.55699, F.Model =7.3343
	Pairwise comparisons	Swab Vs Tissue	0.006281157
		Swab Vs NP40 0.01%	0.008723829
		Swab Vs NP40 0.1%	0.006281157
		Swab Vs Tween 0.01%	0.006281157
		Swab Vs Tween 0.1%	0.007613523
		Swab Vs Tween 1%	0.006281157
		Tissue Vs NP40 0.01%	0.006281157
		Tissue Vs NP40 0.1%	0.006281157
		Tissue Vs Tween 0.01%	0.006281157
		Tissue Vs Tween 0.1%	0.006281157
		Tissue Vs Tween 1%	0.006281157
		NP40 0.01% Vs NP40 0.1%	0.006281157
		NP40 0.01% Vs Tween 0.01%	0.328015952
		NP40 0.01% Vs Tween 0.1%	0.015353938
		NP40 0.01% Vs Tween 1%	0.009663318
		NP40 0.1% Vs Tween 0.01	0.455383848
		NP40 0.1% Vs Tween 0.1%	0.516450648
		NP40 0.1% Vs Tween 1%	0.057577268
		Tween 0.01% Vs Tween 0.1%	0.455383848
		Tween 0.01% Vs Tween 1%	0.010468594
		Tween 0.1% Vs Tween 1%	0.133012727

Supplementary Table S3. Benjamini Hochberg corrected Wilcoxon p-values for comparisons between Reads, Chao 1, Inverse Simpson and Shannon diversity indexes and for PERMANOVA comparisons shown in the PCoA presented in Figure 3.

Comparison 1	Comparison 2	Reads Wilcoxon	Chao1 Wilcoxon	Inverse Simpson Wilcoxon	Shannon Wilcoxon	PERMANOVA
1.00E+08	1.00E+07	0.46	**0.003**	**0.048**	0.045	0.779
1.00E+08	1.00E+06	0.46	**0.003**	0.818	0.045	0.779
1.00E+08	1.00E+05	**0.004**	**0.003**	**0.023**	0.003	**0.006**
1.00E+08	1.00E+04	**0.004**	**0.003**	**0.005**	0.003	**0.010**
1.00E+08	1.00E+03	**0.004**	**0.003**	**0.005**	0.003	**0.006**
1.00E+08	1.00E+02	**0.004**	**0.003**	**0.005**	0.003	**0.006**
1.00E+07	1.00E+06	0.066	**0.003**	**0.048**	0.003	0.779
1.00E+07	1.00E+05	**0.004**	**0.003**	**0.015**	0.003	**0.006**
1.00E+07	1.00E+04	**0.004**	**0.003**	**0.005**	0.003	**0.006**
1.00E+07	1.00E+03	**0.004**	**0.003**	**0.005**	0.003	**0.006**
1.00E+07	1.00E+02	**0.004**	**0.003**	**0.005**	0.003	**0.008**
1.00E+06	1.00E+05	**0.004**	**0.003**	**0.048**	0.006	**0.006**
1.00E+06	1.00E+04	**0.004**	**0.003**	**0.005**	0.003	**0.006**
1.00E+06	1.00E+03	**0.004**	**0.003**	**0.005**	0.003	**0.006**
1.00E+06	1.00E+02	**0.004**	**0.003**	**0.005**	0.003	**0.006**
1.00E+05	1.00E+04	0.651	0.734	**0.036**	0.003	0.232
1.00E+05	1.00E+03	0.699	0.28	**0.023**	0.019	0.136
1.00E+05	1.00E+02	0.46	**0.02**	**0.005**	0.003	**0.009**
1.00E+04	1.00E+03	0.46	0.435	0.265	0.189	0.779
1.00E+04	1.00E+02	0.699	0.051	**0.008**	0.006	0.232
1.00E+03	1.00E+02	0.46	0.818	0.414	0.310	0.248

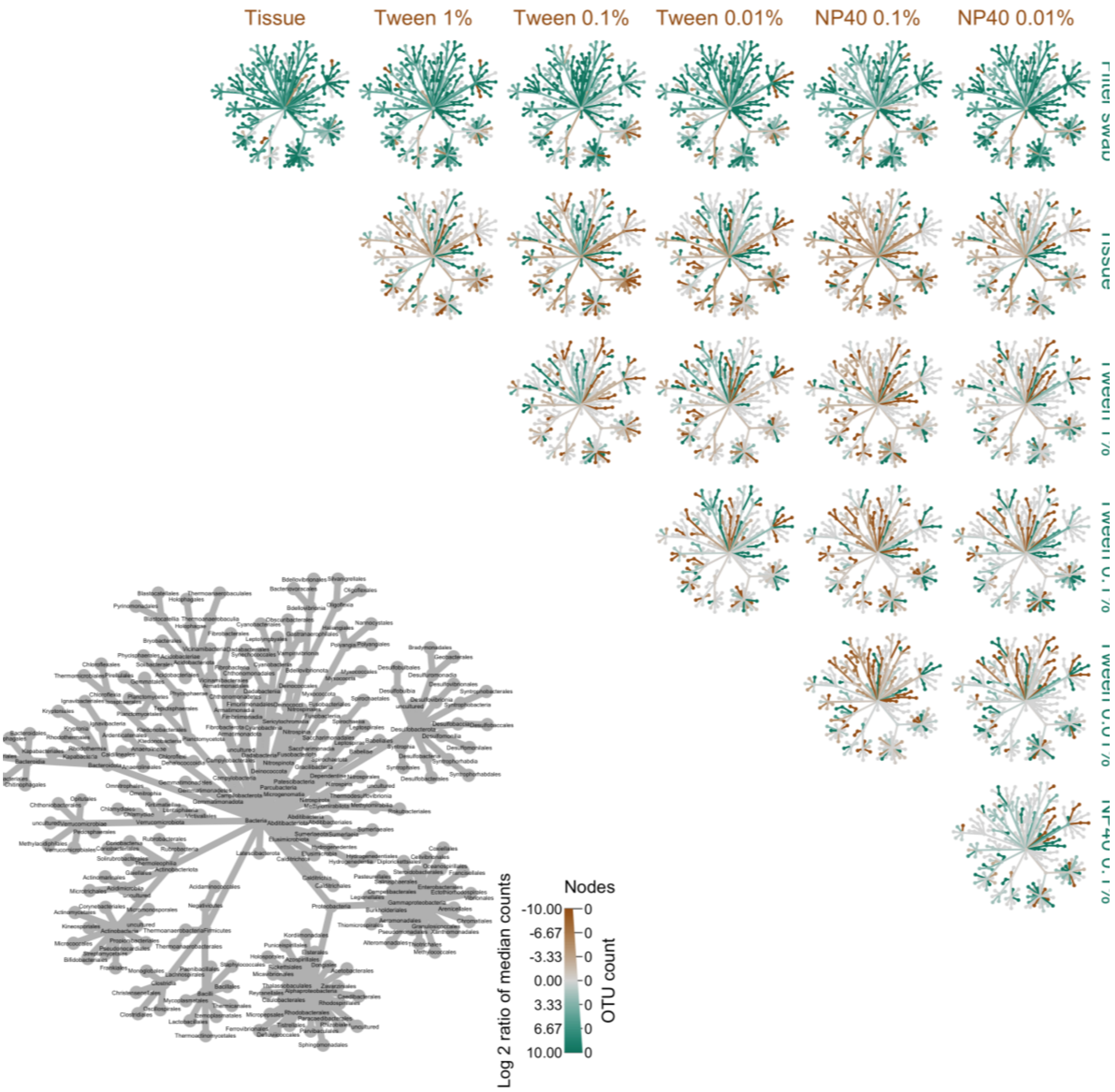

Supplementary Figure S1. Comparisons of significant differences in bacterial phylotype composition between samples. Comparisons are made using pair-wise comparisons between the samples named along the top and the samples named down the side. Each taxon is coloured based upon the log-2 ratio of reads observed in each group based upon the median proportion. Significant differences are coloured based on Wilcox rank-sum test followed by a Benjamini Hochberg correction.

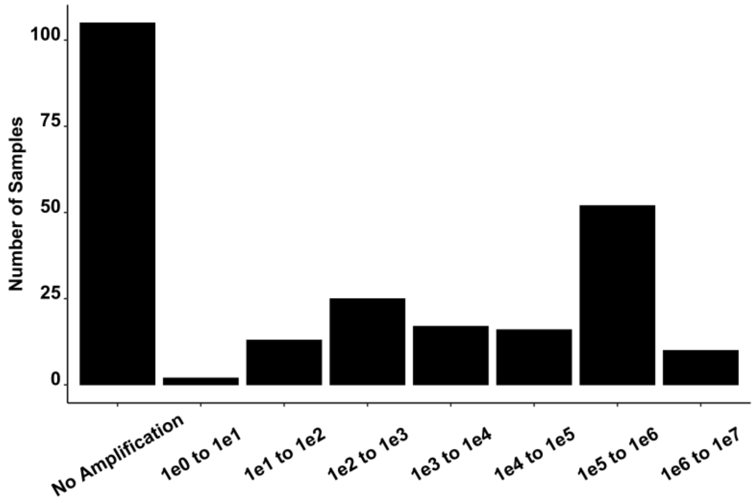

Supplementary Figure S2. Quantification of 16S rRNA copy numbers from a recent trial in our lab. Sample number in each group is presented as a total number and as a percentage of the total.

Supplementary Table S4. Quantification of 16S rRNA copy numbers from a recent trial in our lab. Sample number in each group is presented as a total number and as a percentage of the total.

Group	Number of samples	Percentage of total samples collected
**No Amplification**	105	44%
**1e0 to 1e1**	2	1%
**1e1 to 1e2**	13	5%
**1e2 to 1e3**	25	10%
**1e3 to 1e4**	17	7%
**1e4 to 1e5**	16	7%
**1e5 to 1e6**	52	22%
**1e6 to 1e7**	10	4%

Supplementary Table S5. An example of 20 samples selected from the highest copy number samples from a recent trial are presented with the extracted DNA concentration as measured by Qubit and 16S copy number as quantified by qPCR. Copy number per 1 nanogram of DNA and per 1 uL is presented. A comparison between total copy number in a preparation using 20ng and no normalisation is shown against the theoretical maximum copies that could be included. Samples with higher than 1e5 in the initial step of library preparation are shown in bold.

Sample	Qubit Conc ng/ul	16s Copy Number in 1ng	16s Copy Number in 1 uL of original sample	16S Copy number in 10ngs of DNA	Copies in 4.2ul (Max Reaction Volume)
1	23.1	4764	110037	47635	**462155**
2	10.4	1562	16245	15620	68228
3	17.5	2977	52089	29765	**218773**
4	17.6	577	10159	5772	42669
5	12.1	767	9281	7670	38979
6	20.2	4626	93440	46258	**392449**
7	18.1	2315	41892	23145	**175948**
8	13.9	641	8915	6414	37443
9	4.27	4900	20923	48999	87875
10	22.5	2977	66971	29765	**281279**
11	18.6	717	13342	7173	56038
12	18.4	5215	95947	52145	**402977**
13	20.9	693	14487	6932	60845
14	3.93	6857	26948	68569	**113180**
15	22.7	1205	27348	12048	**114861**
16	39.7	1832	72730	18320	**305468**
17	28.8	1300	37442	13001	**157257**
18	7.59	1294	9821	12940	41250
19	4.47	11660	52120	**116600**	**218904**
20	25.3	1614	40828	16138	**171477**

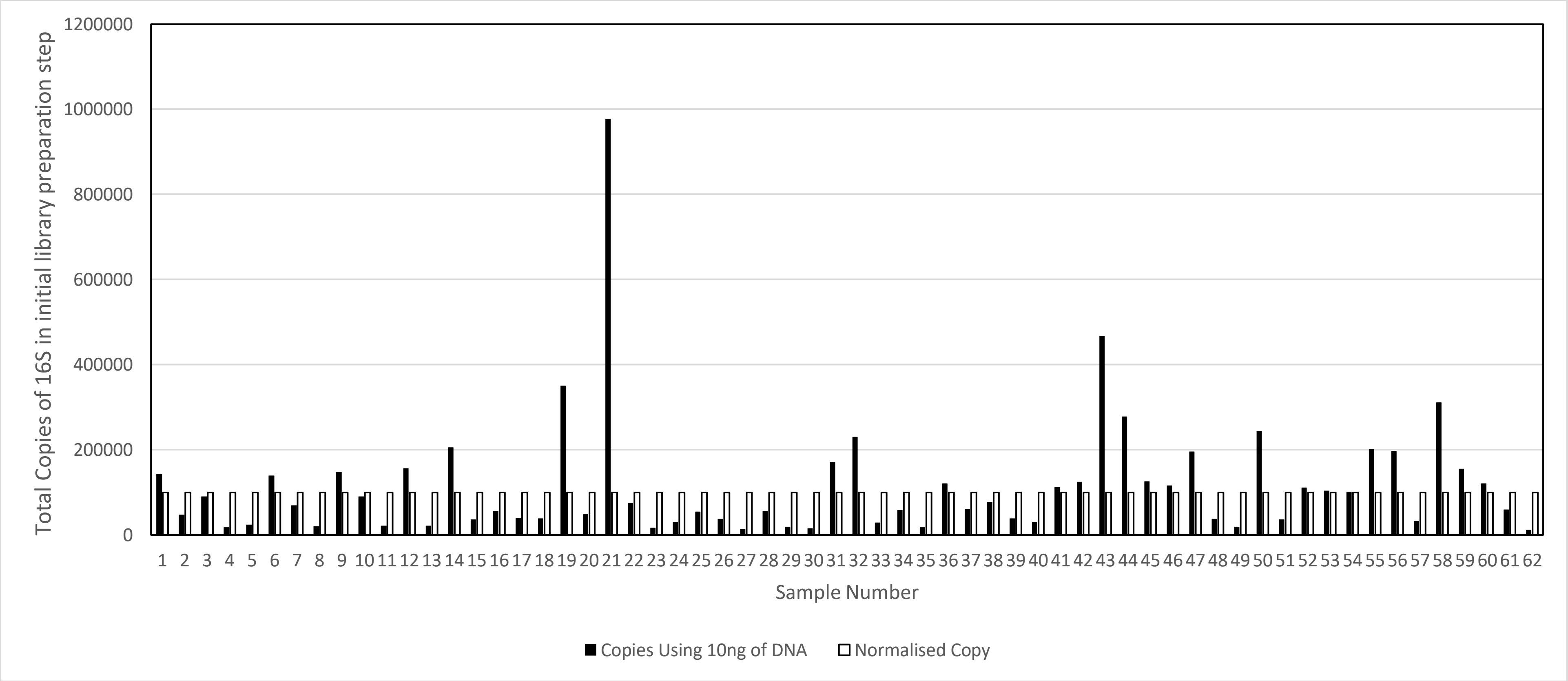

Supplementary Figure S3. Total copies of 16S present in a non-normalised library preparation using 10ng in triplicate versus normalised library preparation based upon an input of 1e5 copies

Supplementary Table S6. Table of primers and probes.

Primer	Primer Sequence (5'- 3')	Target	Size	Reference
V3-4 [314F]	CCTACGGGNGGCWGCAG	16S rDNA V3-4	466	
V3-4 [805R]	GACTACHVGGGTATCTAATCC			
V3_V4_TaqMan	ATTACCGCGGCTGCTGG			
16s_V4F	TCGTCGGCAGCGTCAGATGTGTATAAGAGACAGAYTGGGYDTAAAGNG	16S rDNA V4	~245bp	Ma *et al.* 2017
16s_V4R_1	GTCTCGTGGGCTCGGAGATGTGTATAAGAGACAGTACCRGGGTHTCTAATCC			
16s_V4R_2	GTCTCGTGGGCTCGGAGATGTGTATAAGAGACAGTACCAGAGTATCTAATTC			
16s_V4R_3	GTCTCGTGGGCTCGGAGATGTGTATAAGAGACAGCTACDSRGGTMTCTAATC			
16s_V4R_4	GTCTCGTGGGCTCGGAGATGTGTATAAGAGACAGTACNVGGGTATCTAATC			
V4_Sp1_Mix_F	TCGTCGGCAGCGTCAGATGTGTATAAGAGACAGGAYTGGGYDTAAAGN*G			
V4_Sp1_Mix_R1	GTCTCGTGGGCTCGGAGATGTGTATAAGAGACAGTCAGTACCRGGGTHTCTAATC*C			
V4_Sp1_Mix_R2	GTCTCGTGGGCTCGGAGATGTGTATAAGAGACAGTCAGTACCAGAGTATCTAATT*C			
V4_Sp1_Mix_R3	GTCTCGTGGGCTCGGAGATGTGTATAAGAGACAGTCAGCTACDSRGGTMTCTAAT*C			
V4_Sp1_Mix_R4	GTCTCGTGGGCTCGGAGATGTGTATAAGAGACAGTCAGTACNVGGGTATCTAAT*C			
V4_Sp4_Mix_F	TCGTCGGCAGCGTCAGATGTGTATAAGAGACAGAGTCAYTGGGYDTAAAGN*G			
V4_Sp4_Mix_R1	GTCTCGTGGGCTCGGAGATGTGTATAAGAGACAGCTACCRGGGTHTCTAATC*C			
V4_Sp4_Mix_R2	GTCTCGTGGGCTCGGAGATGTGTATAAGAGACAGCTACCAGAGTATCTAATT*C			
V4_Sp4_Mix_R3	GTCTCGTGGGCTCGGAGATGTGTATAAGAGACAGCCTACDSRGGTMTCTAAT*C			
V4_Sp4_Mix_R4	GTCTCGTGGGCTCGGAGATGTGTATAAGAGACAGCTACNVGGGTATCTAAT*C			
AS_cldn18_F	AGCTACATGTGATTGGCATCG	claudin 18	241	
AS_cldn18_R	TCTTTCTACCTCAGTTATGGGATG			
OM_NaKa1a_F	ACATTGAGTGATCCTGGGACG	NaKa1a	173	
OM_NaKa1a_R	ATGTAAGATTGGGAGTGCGGT			
